# Add-On Treatment with *Passiflora incarnata L., herba,* during Benzodiazepine Tapering in Patients with Depression and Anxiety: A Real-World Study

**DOI:** 10.3390/ph16030426

**Published:** 2023-03-10

**Authors:** Raffaella Zanardi, Matteo Carminati, Valentina Fazio, Melania Maccario, Greta Verri, Cristina Colombo

**Affiliations:** 1 Department of Clinical Neurosciences, Vita-Salute San Raffaele University, 20132 Milan, Italy; 2 Department of Clinical Neurosciences, Mood Disorder Unit, IRCCS San Raffaele Institute, 20127 Milan, Italy

**Keywords:** benzodiazepines discontinuation, *Passiflora incarnata*, depression, anxiety, real-world study, pharmacological treatment, GABA

## Abstract

Chronic and inappropriate benzodiazepine intake represents an important health and social concern worldwide. The aim of our study was to investigate the effectiveness of *P. incarnata L., herba,* in reducing benzodiazepine misuse in a real-world population of depressed and anxious patients in a long-term treatment with benzodiazepines. We conducted a retrospective naturalistic study on 186 patients undergoing benzodiazepine downtitration, 93 with the addition of a dry extract of *P. incarnata L., herba* (Group A), and 93 without any add-on treatment (Group B). Regarding the benzodiazepine dosage variation in the two groups, a repeated measure ANOVA showed a significant effect of time (*p* < 0.001), group (*p* = 0.018), and time x group interaction (*p* = 0.011). We found a significantly higher rate, i.e., of 50%, reduction in Group A vs. Group B at 1 month (*p* < 0.001) and at 3 months (*p* < 0.001) and complete benzodiazepine discontinuation at 1 month (*p* = 0.002) and at 3 months (*p* = 0.016). Our findings suggest the role of *P. incarnata* as an effective add-on treatment during benzodiazepine tapering. These findings highlight the need for further studies to better investigate the promising properties of *P. incarnata* in the management of such a relevant clinical and social issue.

## 1. Introduction

Benzodiazepines (BDZs) are widely prescribed for a variety of clinical conditions [1], including anxiety disorders, mood disorders, and insomnia. The psychopathology of all of these conditions is classically explained with the monoaminergic theory. More recent studies suggest that a disbalance in the glutamate/γ-aminobutirric acid (GABA) neurotransmission is also involved [2,3]. In particular, GABAergic transmission plays an important role in the control of hippocampal neurogenesis and neural maturation, which explains the cognitive and affective symptoms of these conditions [4,5,6].

Due to their rapid effectiveness and low toxicity, BDZs often represent the first treatment administered by general practitioners and other specialists, also outside of psychiatric setting [6,7]. However, in the last decades, physicians’ enthusiasm has been curbed by emerging concerns about abuse, dependence, higher risk of falls, and possible cognitive disturbances [6,8,9,10,11]. Both chronic and inappropriate benzodiazepine (BDZ) intake represent, indeed, an important health and social concern worldwide [7], leading to an increased risk of oversedation, cognitive and psychomotor impairment, and physical injuries [12].

Despite the aforementioned risks, BDZs are still one of the most prescribed classes of medications [13]. The prevalence of benzodiazepine prescription in the general population is between 2% and 3% in most European countries [14] and even 4% to 5% in the USA [13]. Specifically, the long-term therapeutic use of BDZs has been subject to debate during the last decades [8], with only limited evidence suggesting long-term efficacy in specific disorders, such as generalized anxiety disorder [15], panic disorder [16,17], and social phobia [18]. On the other hand, the prevalence of these disorders among the long-term BDZ users is quite low [19], and current guidelines strongly recommend selective serotonin reuptake inhibitors (SSRIs) over BDZs as the best choice for the treatment of all the anxiety disorders, together with cognitive–behavioral therapy (CBT) [20]. Even though BDZs are recommended by current prescribing guidelines only as augmentative agents, especially to mitigate the adverse side effects during the initial weeks of therapy with antidepressants, BDZs are prescribed just as frequently as SSRIs and SNRIs (serotonin and noradrenaline reuptake inhibitors), though primarily by non-psychiatrists [21].

BDZs cause a positive allosteric modulation of GABA_A_ receptors [22], amplifying the inhibiting effect of the endogenous neurotransmitter and thereby explicating sedative, anxiolytic, and hypnotic effects. This action is characterized by a rapid onset, giving the patients an immediate sense of relief from symptoms, although it does not represent a long-term therapeutic effect on neurobiological alterations of anxiety and mood disorders. BDZs also produce a GABAergic signaling increase in the ventral tegmental area (VTA) and nucleus accumbens (NAc) [23], inducing an enhancement of reward circuitry and therefore causing symptoms of craving when the drug is suspended. Due to these phenomena, BDZs can easily be misused by deliberate or unintentional abusers [13]. The first group includes both users of BDZs as their primary drug of abuse or polyabusers, and they were not considered for this study. The second one, unintentional abusers, includes patients taking benzodiazepines for a long course of time, under medical prescription, who extend the period and dosage on their own will to avoid withdrawal symptoms or to maintain the same subjective effects [13,24,25,26,27].

BDZ discontinuation is, indeed, made difficult by a variety of symptoms characterizing the so-called *discontinuation syndrome*. They include the relapse of initial anxiety symptoms, a rebound or intensification of the original symptoms, or an actual withdrawal syndrome, consisting of irritability; psychomotor agitation; and physical symptoms such as headache, sweating, palpitations, nausea, vomiting, and rarely muscular pain and seizures [22,23,27,28,29,30]. Since BDZs are so widely prescribed by physicians, patients often have a false sense of safety regarding their use. Therefore, a majority of unintentional abusers are scarcely aware of their BDZ dependence until they try to discontinue the drug [13], making the real prevalence of the phenomenon difficult to estimate.

In order to avoid rebound or withdrawal symptoms, many strategies have been studied to implement effective BDZ tapering programs [31,32]. The most common clinical approaches are a gradual tapering of the current molecule or a switch to a long-acting BDZ in order to facilitate a smoother discontinuation. A common schedule consists of a 25% reduction of dosage every 2 weeks and a slower taper of 12.5% every 2 weeks near the end of stopping [33]. However, tapering alone is not effective in about one-third of patients because of a rebound of anxiety and demoralization [12] and/or the occurrence of withdrawal syndrome.

Research on non-benzodiazepine GABAergic medications, such as pregabalin [34,35,36] or gabapentin [37], has been conducted for BDZ tapering and withdrawal management, with conflicting results. Other adjuvant medications were studied as augmentation strategies during BDZ tapering in order to facilitate the outcome and reduce the negative symptoms [38,39]. Negative results were reported for the β-blocker propranolol [40], for progesterone [41], for the 5-hydroxy-tryptamine-3 antagonist ondansetron [42], and for the tricyclic antidepressant dothiepin [43]. The use of buspirone as an augmentation strategy gave mixed results [44,45], while imipramine [46], carbamazepine [47], valproate [48], and trazodone [48] were found to be effective in BDZ tapering when compared to a placebo. Research has also been carried out on melatonin and herbal remedies that show anxiolytic and hypnotic properties, such as *Valeriana officinalis*, *Melissa officinalis*, and *Eschscholzia californica Cham*, whose effectiveness is, however, limited [49].

*Passiflora incarnata*, a species of the *Passiflora genus*, has well-documented therapeutic properties and has been used for medicinal purposes for centuries as a remedy for diarrhea, painful menstruation, insomnia, convulsions, or anxiety [50]. Flowers and fruits are a source of alkaloids, flavoinoids, phenols, and cyanogenic glycosides. There is evidence of its effect as a modulator of the GABA system, binding to the GABA site on the GABA_A_ post synaptic receptor, and acting as an antagonist of GABA_B_ pre-synaptic receptor, a modulator of the neurotransmitter release [51,52,53]. Some studies suggest that modulators of the GABA_B_ receptor may have an anxiolytic effect and be helpful in the treatment of substance-use disorders [34]. *Passiflora incarnata L., herba*, as a dry extract, was recently approved by European Medicines Agency (EMA) as a medicinal product and was found to be effective in reducing anxiety symptoms [54].

The aim of our study was to investigate the effectiveness of *P. incarnata L., herba,* as a tool in reducing BDZ misuse, facilitating the withdrawal regimen in a real-world population of depressed and anxious patients in a long-term treatment with BDZs. Our findings suggest the role of *P. incarnata* as an effective add-on treatment during BDZ tapering. Specifically, the better management of withdrawal symptoms and anxiety rebound allowed a faster reduction of BDZs in patients taking *P. incarnata* compared to patients undertaking a classical tapering program. We hypothesized a correlation between the known pharmacological effects on GABA_A_ and GABA receptors of flavonoid fraction of *P. incarnata* and the observed clinical outcome.

## 2. Materials and Methods

Over an 18-month period (from July 2021 to December 2022), we conducted a retrospective and naturalistic study on euthymic outpatients with a diagnosis of anxiety or depression and chronically taking BDZs. All patients referred to Mood Disorder Unit at San Raffaele Hospital in Milan, Italy, and represented a real-world target population.

### 2.1. Participants

We collected 93 euthymic patients undergoing a BDZ tapering regimen and taking a dry extract of *Passiflora incarnata L., herba,* for the management of anxiety symptoms, at a daily fixed dosage ranging from 200 mg to 600 mg depending on clinical evaluation. These patients were compared with a sex, age (+/− 5 years), and diagnosis matched group of 93 subjects randomly selected from our pool of outpatients undergoing a traditional BDZ tapering program, based on a slow weekly reduction of BDZ dosage, without any add-on treatment.

Inclusion criteria were as follows: age >17 years; fulfilling the Diagnostic and Statistical Manual of Mental Disorders 5th edition (DSM-5) [55] for any of the featured anxiety or depressive disorders; clinical remission of the depressive episode (Hamilton Depression Rating Scale 21 items <17) [56]; mild anxiety symptoms (Hamilton Anxiety Rating Scale ≤17) [57]; chronic BDZ consumption (>6 weeks); and treatment with a SSRI, SNRI, or other antidepressants. We excluded patients diagnosed with psychotic disorders, patients in treatment with antipsychotic or mood stabilizers, and patients affected by any substance-use disorder, apart from benzodiazepine misuse and abuse.

### 2.2. Passiflora incarnata L., herba

This drug was approved in Italy by AIFA (Agenzia Italiana del FArmaco) as a medical product since November 2020 (Tractana^®^), is available in 200 mg tablets (maximum recommended daily dosage of 800 mg), and is widely prescribed in our center for the management of mild anxiety symptoms and sleep disturbances. The most common tapering schedule in our outpatient facility consists of a 25% reduction of dosage every 2 weeks and a slower taper of 12.5% every 2 weeks near the end of stopping. This scheme is, however, tailored according to each patient based on tolerance and anxiety rebound. No patients had ever taken Tractana^®^ before the beginning of the observational period.

### 2.3. Data Collection

At baseline, we recorded sociodemographic data, current treatments, and diagnosis. BDZ dosages for each patient at each time-point were reported as diazepam milligram equivalents (thereafter referred as mg-equiv).

Anxious and depressive symptomatology was investigated both objectively and subjectively, using Hamilton Anxiety Rating Scale (HARS) [57], Hamilton Depression Rating Scale (HDRS) [56], Beck Anxiety Inventory (BAI) [58], and Beck Depression Inventory (BDI) [59].

Anamnestic data were collected from patients’ medical records at baseline (T0), while psychometric assessments and BDZ assumption were collected at T0, after 1 month (T1), and three months (T2) from the start of BDZ reduction.

Anamnestic data and psychometric assessments were collected by a trained psychiatrist. The study, approved by the Ethical Committee of the Hospital, was conducted in accordance with the Declaration of Helsinki, and all patients’ data were treated confidentially and anonymously.

### 2.4. Statistical Analyses

All statistical analyses were performed using JASP [60] (version 0.16.4) computer software; tables and graphs were obtained by JASP or Microsoft Excel [61] (version 15.59) software. All tests were two-tailed, with a statistical significance level set at <0.05. Continuous variables are expressed as mean ± standard deviation (SD), while categorical variables are reported as numerosity and percentages. Using the Shapiro–Wilk normality test, we verified the non-normal distribution of continuous variables in the global sample and both groups. Friedman’s non-parametric version of repeated measures ANOVA (rANOVA) was performed on psychometric tests, as well as on mean BDZ dosage, to assess the progression of the withdrawal regimen. An analysis of covariance (ANCOVA) was performed to investigate the relationship between dry extract of *P. incarnata L., herba,* and the percent reduction of BDZ over time, correcting for age, sex, diagnosis, and initial BDZ dosage. Chi-squared tests were performed to compare categorical variables between groups.

We collected all patients meeting inclusion criteria and referring to our outpatient facility during the observation period. A power analysis based on the collected data was performed to evaluate the chances of detecting a significative group difference in BDZ dose reduction.

## 3. Results

The whole sample consisted of 186 patients undergoing a protocol of BDZ downtitration, 93 with the addition of a dry extract of *Passiflora Incarnata L., herba* (Group A, mean age: 54.6 ± 16.4), and 93 without any add-on treatment (thereafter referred as Group B; mean age: 51.7 ± 13.1).

We found no differences between the two samples in age, sex, occupancy level, years of education, and diagnosis. No significant differences in the psychometric baseline scores were found between the two groups. The sociodemographic and clinical features of the sample are reported in Table 1.

**Figure 1:**
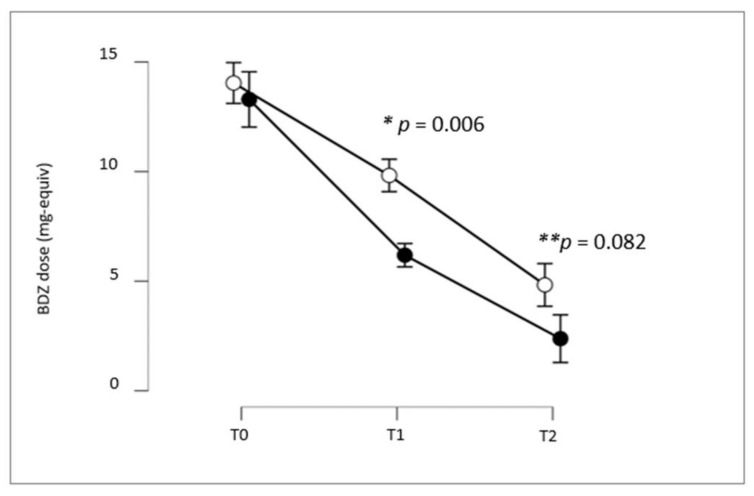
Means (black and white circles are, respectively, Group A and Group B) and 95% confidence intervals (vertical bars) of the BDZ dosage (mg-equiv) at baseline/pretreatment (T0), at the end of the first month of treatment (T1), and at the end of the third month of treatment (T2). * rANOVA post hoc comparisons Group A vs. Group B at T1; ** rANOVA post hoc comparisons Group A vs. Group B at T2.

We performed a repeated-measures ANOVA to evaluate BDZ dosage (mg-equiv) change in the two groups over time, showing a significant effect of time (*p* < 0.001), group (*p* = 0.018), and time x group interaction (*p* = 0.011), meaning that BDZ dosage significantly changed over time within the two groups, and the pattern of reduction over time did not follow parallel slopes between the two treatment groups, as shown in Figure 1.

Holm’s post hoc comparisons confirmed that BDZ dosage showed a significant decrease within the two groups at each time point (Group A: T0 vs. T1, mean difference: 3.47, *p* = 0.007; T0 vs. T2, mean difference: 10.91, *p* < 0.001; T1 vs. T2, mean difference: 3.81, *p* < 0.001; Group B: T0 vs. T1, mean difference: 4.22, *p* < 0.001; T0 vs. T2, mean difference: 9.21, *p* < 0.001; T1 vs. T2, mean difference: 4.99, *p* < 0.001). Patients treated with *P. incarnata* showed a significantly faster BDZ reduction compared with a standard BDZ tapering regimen (Group B vs. Group A at T0: mean difference: 0.75, *p* = 0.49; at T1: mean difference 3.64, *p* = 0.006; at T2: mean difference 2.45, *p* = 0.082).

Considering the effect of other demographic and clinical variables on the observed BDZ dose change over time, the ANCOVA on the percent BDZ reduction at T1 and T2 showed a significant difference between the two groups at each time point, correcting for age, sex, diagnosis, and starting BDZ dosage, with a significantly greater reduction in the group treated with *P. incarnata L., herba,* at each time point (ΔT0–T1, Group A 62.7.6% vs. Group B: 30.9%, *p* < 0.001; ΔT0–T2: Group A 88.6%, Group B: 66.7%, *p* < 0.001).

We performed a Pearson’s chi-squared evaluating the different rate of 50% BDZ reduction and complete discontinuation between the groups. As shown in Table 2, we found a significantly higher rate of reduction and withdrawal of benzodiazepine in patients taking *P. incarnata* (50% BDZ reduction at 1 month: Group A 76.35%, Group B 26.88%, *p* < 0.001; at 3 months: Group A 95.69%, Group B 72.04%, *p* < 0.001; complete BDZ discontinuation at 1 month: Group A 32.26%, Group B 12.90%, *p* = 0.002; at 3 months: Group A 69.89%, Group B 52.69%, *p* = 0.016).

We performed a repeated-measures ANOVA on each clinical scale (HARS, BAI, HAM-D, and BDI) to evaluate the variation of anxiety symptoms and sleep disturbances during the BDZ tapering program. We found no significant effect of time, group, or time x group on the variation of clinical scores.

**Figure 2:**
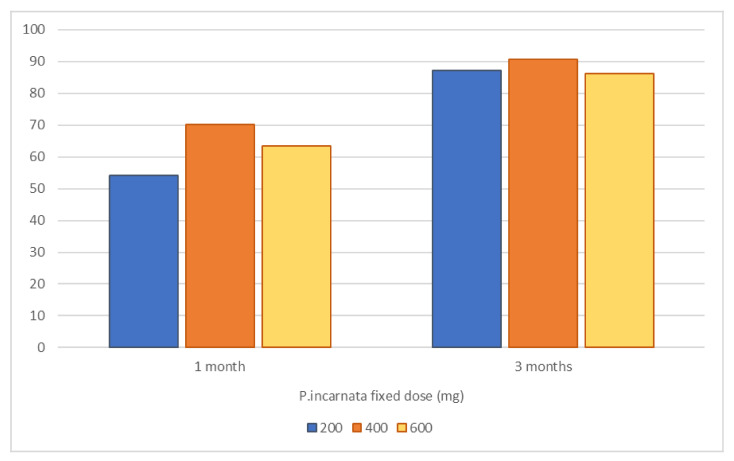
Percent BDZ reduction after 1 month or 3 months from the beginning of the tapering program in patients treated with a dry extract of *P. Incarnata L., herba*.

Finally, selecting only patients from the group treated with *P. incarnata L., herba*, we performed an ANCOVA to investigate the relationship between the *P. incarnata* fixed dosage and the percent BDZ reduction at 1 month and 3 months respectively, correcting for age, sex, diagnosis, and starting BDZ dosage. We found a significant effect of *P. incarnata* dosage on BDZ reduction rate at 1 month (T1, *p* = 0.035), but not significant effect at 3 months (T2, *p* = 0.440), in line with the previous findings. In particular, Holm’s post hoc comparison showed a significant lower rate of BDZ reduction in patients taking 200 mg of dry extract of *P. incarnata L., herba,* than patients taking 400 mg or 600 mg (200 mg vs. 400 mg, mean difference 19.8, *p* = 0.024; 200 mg vs. 600 mg, mean difference 24.3, *p* = 0.024; 400mg vs. 600mg, mean difference 4.5, *p* = 0.619), as shown in Figure 2.

## 4. Discussion

Benzodiazepines (BDZs) are indicated for the short-term treatment of anxious and depressive symptomatology and of sleep disturbances. However, prescriptions are often prolonged even after the remission of anxiety or insomnia, due to the development of physical and psychological dependence [30]. Therefore, the availability of effective tapering protocols is a relevant issue in daily clinical practice, both in psychiatry and general medicine settings [62]. The most common protocol consists of a gradual individualized reduction of BDZ dose in order to minimize withdrawal symptoms [63]. These symptoms may be similar to the ones that led to the initial BDZ prescription, leading patients and caregivers to the wrong supposition that the prolonged assumption is required to avoid relapses or rebound symptoms [38]. This can lead to a prolonged duration of the intake or to a postponement of tapering, with a negative impact in terms of long-term BDZ side effects, and motivation in continuing the reduction to reach complete cessation [12].

Various pharmacological add-on strategies have been investigated to accelerate BDZ reduction, including drugs acting on anxiety and insomnia or treatments targeting psychological craving, such as non-benzodiazepine sedating drugs. However, no drugs are currently recommended for the management of BDZ dependence after long-term use [14].

A dry extract of *P. incarnata L., herba,* has been approved by EMA [54] for the treatment of mild anxiety symptoms. Studies on the pharmacological action of *P. incarnata L., herba,* indicate a possible effect on both anxiety symptoms and craving, suggesting a possible role of this medical product in the management of BDZ discontinuation [52].

Our results, obtained from a sample of euthymic patients who never interrupted BDZ assumption, appear to confirm this hypothesis, showing a more rapid reduction, as compared to classical tapering, when using add-on therapy with *P. incarnata L., herba*.

In particular, the group treated with *P. incarnata* showed a significantly greater reduction of BDZ mean dosage both after 1 and 3 months from the beginning of the tapering program. This difference between the groups is more relevant at the first time point, suggesting an effect of *P. incarnata* as an accelerator of BDZ tapering. This finding is confirmed by the different rate of 50% reduction and complete cessation of BDZs between the two groups, showing a significant difference at each time point, to a greater degree at 1 month than 3 months. The difference observed between the groups appeared to not be correlated with recorded clinical and demographic variables, including sex, age, diagnosis, or starting BDZ dosage. During the observation period, anxiety and depression symptomatology scores, as assessed by clinical scales, did not significantly change in both patients group.

From a pharmacodynamic point of view, *P. Incarnata* acts as a modulator of the GABA system [52,53,64]; in particular, the benzoflavonoid fraction acts as an agonist of the GABA_A_ post-synaptic receptor and as an antagonist of the GABA_B_ pre-synaptic receptor, thus modulating GABA pre-synaptic release and action on the post-synaptic neuron.

The effect on the GABA_A_ receptor is similar to BDZ pharmacological effect, suggesting a possible role of *P. incarnata* as an alternative to BDZs for the management of anxiety symptoms. This effect is surely useful in BDZ tapering programs, since it may help in reducing the rebound of anxiety symptoms, one of the main clinical manifestations of BDZ withdrawal syndrome.

The antagonism on pre-synaptic GABA_B_ receptor may also explain the effectiveness of *P. incarnata* in BDZ reduction, since this action has been supposed to have a clinical effect on craving reduction. Experimental clinical studies in animal and humans suggest that modulators of the GABA_B_ receptor may have an anxiolytic effect, indeed, and might be helpful in the treatment of substanc- use disorders [65]. This efficacy in terms of control of BDZ dependence has been confirmed by a study on mice treated with diazepam and/or the flavonoid fraction of *P. incarnata*. This extract was shown to significantly reduce the withdrawal symptoms of diazepam dependence in a dose-dependent manner [66].

These data are in line with our findings about the relationship between the clinical effect and dosage of *P. incarnata*. Specifically, we found a significant difference between patients taking 200 mg of dry extract of *P. incarnata L., herba,* and 400 or 600 mg, respectively. This difference, observed at 1 month after the beginning of the tapering program, was not confirmed at 3 months, thus validating the hypothesized accelerating role of *P. incarnata* in BDZ reduction.

The efficacy of *P. incarnata* in the management of anxiety was investigated in trials on humans, as well, but the scarcity of placebo-controlled studies is a critical lack. However, a double-blind placebo-controlled preliminary study on the effect of *P. incarnata* herbal tea on subjective sleep quality showed short-term subjective sleep benefits for healthy adults with mild fluctuations in sleep quality. The authors stated that one possible explanation for this result could be that the participants held preconceived expectances regarding the effects of *P. incarnata*, meaning they were able to recognize *P. incarnata* as the true condition and reported improvements in their sleep quality [67]. This is not the case in our study, since patients referring to our outpatient facility did not have a previous experience with *P. incarnata* and did not have expectancies regarding its possible positive effect. On the other hand, they already had a positive subjective experience towards BDZs, since they could relate the assumption of them with the remission of their anxiety or depressive symptoms.

A pilot double-blind randomized controlled trial showed similar efficacy of *P. incarnata* extract and oxazepam for the treatment of generalized anxiety disorder, suggesting that *P. incarnata* may be an effective drug for the management of anxiety symptoms. In particular, oxazepam showed a more rapid onset of action but a significantly greater impairment of performance compared to *P. incarnate* [68]. Although this study was conducted on a small sample of patients, the lower incidence of impairment of performance could be an important advantage of P. incarnata extract compared to BDZs. This finding is particularly relevant in the context of BDZ discontinuation, since performance impairment is the main concern about long-term BDZ treatment.

In our study, a possible placebo effect should not be neglected in the group of patients taking *P. incarnata*. The fact to not interrupt any drug assumption may play a role in maintaining well-being, also because patients who previously tried BDZ discontinuation may have experienced a rebound of symptoms, leading to a fear of drug discontinuation. Moreover, many patients may associate the ritual of taking medicine with a positive healing effect. The EMA approval of a dry extract of *P. incarnata L., herba,* as a medical product could enhance this effect, since some patients who are used to taking antidepressants in psychiatric settings may not trust the beneficial effects of herbal products or nutraceuticals, preferring a prescription of molecules with stronger preclinical and clinical evidence of efficacy. The placebo effect may not have been isolated from the pharmacological effect, given the retrospective observational nature of the study. However, this effect may also represent a useful tool in managing BDZ tapering in a subgroup of patients.

To our knowledge, this is the first study to evaluate the effectiveness of *P. incarnata L., herba,* in reducing BDZ misuse in people living with anxiety and depressive disorders. The non-randomized nature of the study offers a representative picture of the real-world situation, also considering the numerical relative consistency of the sample.

## 5. Limitations and Strengths

The study has some limitations. The first is the retrospective and observational nature of this study, which did not include a placebo-controlled group. Moreover, the data collection was based on medical records, thus leading to a lack of relevant clinical information, such as the presence of personality disorders or a history of BDZ abuse. Secondly, the short follow-up period (3 months) did not allow us to draw any firm conclusion regarding the sustained effect on BDZ interruption. In the period of observation, we did not observe any relapse or dropout. No significant withdrawal effects were recorded. In line with previous studies and reviews on the topic, we used different outcome measures to evaluate the effectiveness of BDZ reduction, including the rate of complete BDZ cessation; mean dose reduction; rebound of anxiety and demoralization symptoms, as measured by clinical rating scales; and adverse events, defined as any undesirable medical event experienced by patients [38] and collected from medical records. However, no craving rating scale was administered. Given the retrospective nature of the study, we could assess the compliance to the treatment only by asking patients themselves and caregivers.

On the other hand, our work has important strengths. The most relevant is its novelty, since only a few studies were conducted on the clinical effect of *P. incarnata* on large population samples. Moreover, the observational nature of the study leads to a narrowing of the gap with clinical reality, giving information about the real-world feasibility of the treatment.

## 6. Conclusions and Future Directions

Our findings support the effectiveness of a dry extract of *P. incarnata L., herba,* as an add-on treatment during BDZ tapering in patients with anxiety or depression. More specifically, we observed a more rapid reduction of BDZs, as compared to classical tapering, when using add-on therapy with *P. incarnata*. The absence of side effects and adverse events confirmed the safety of *P. incarnata* in a real-world population. These preliminary findings need to be confirmed by specifically designed clinical trials. Further studies may be helpful to better investigate the promising properties of *P. incarnata* in the management of relevant clinical issues, such as anxiety disorders and addiction, that are classically known to benefit from GABAergic treatments.

## Figures and Tables

**Table 1:** Sociodemographics and clinical data of the sample.

	Group A (*P. incarnata*) (*n* = 93)	Group B (*n* = 93)
Age (years)	54.6 ± 16.4	51.7 ± 13.1
Sex ratio (M/F)	27/66 (29.0%/71.0%)	35/58 (37.6%/62.4%)
Education (years)	13.4 ± 3.55	12.6 ± 3.43
Occupation (U/E)	13/80 (13.9%/86.1%)	17/76 (18.3%/81.7%)
Status (single/coupled)	23/70 (24.7%/75.3%)	24/69 (25.8%/74.2%)
Diagnosis (depression/anxiety)	43/50 (46.2%/53.8%)	48/45 (51.6%/48.4)
**Baseline clinical measurements**		
HARS	6.43 ± 4.76	6.34 ± 4.77
BAI	5.75 ± 4.68	5.59 ± 4.63
HDRS	4.69 ± 3.73	4.64 ± 3.68
BDI	4.89 ± 3.85	4.85 ± 3.83

Abbreviations: M, male; F, female; U, unemployed; E, employed; HARS, Hamilton Anxiety Rating Scale; BAI, Beck Anxiety Inventory; HDRS, Hamilton Depression Rating Scale; BDI, Beck Depression Inventory.

**Table 2:** Percentage of patients achieving 50% reduction or complete BDZ discontinuation after 1 and 3 months in the two groups.

	Group A *P. incarnata L., herba*	Group B No Add-On Treatment	*p*-Value *
**50% BDZ reduction**			
1 month	76.35%	26.88%	<0.001
3 months	95.69%	72.04%	<0.001
**Complete BDZ discontinuation**			
1 month	32.26%	12.90%	0.002
3 months	69.89%	52.69%	0.016

* A Pearson’s chi-squared was performed, and *p*-values are reported in the table.

## Data Availability

Data is contained within the article.
